# Cardiac Arrhythmias and Their Management: An In-Depth Review of Current Practices and Emerging Therapies

**DOI:** 10.7759/cureus.66549

**Published:** 2024-08-09

**Authors:** Anmol K Nagpal, Aditya Pundkar, Akhilesh Singh, Charuta Gadkari

**Affiliations:** 1 Emergency Medicine, Jawaharlal Nehru Medical College, Datta Meghe Institute of Higher Education and Research, Wardha, IND; 2 Orthopedics, Jawaharlal Nehru Medical College, Datta Meghe Institute of Higher Education and Research, Wardha, IND

**Keywords:** emerging therapies, electrophysiology, antiarrhythmic drugs, catheter ablation, atrial fibrillation, cardiac arrhythmias

## Abstract

Cardiac arrhythmias encompass a range of conditions characterized by abnormal heart rhythms, affecting millions globally and significantly contributing to morbidity and mortality. This review provides a comprehensive analysis of the current practices and emerging therapies in managing cardiac arrhythmias, covering their definition, classification, epidemiology, and the critical importance of effective management. It explores the pathophysiology underlying various arrhythmias, including the mechanisms of arrhythmogenesis, such as re-entry, automaticity, and triggered activity. The review details the latest diagnostic tools, including ECG, Holter monitoring, and electrophysiological studies, and discusses the clinical presentation of different arrhythmias, from supraventricular to ventricular types and bradyarrhythmias. We examine current pharmacological and non-pharmacological treatment strategies, such as antiarrhythmic drugs, catheter ablation, and device therapy, highlighting their efficacy and limitations. Furthermore, the review delves into emerging therapies, including advanced catheter ablation techniques, novel antiarrhythmic agents, gene therapy, and innovative device technologies like leadless pacemakers and subcutaneous implantable cardioverter-defibrillators (ICDs). Special considerations for managing arrhythmias in diverse populations, including pediatrics, the elderly, and pregnant women, are discussed. Additionally, the review explores future directions in arrhythmia management, emphasizing personalized medicine, artificial intelligence applications, and the integration of advanced technologies in diagnosis and treatment. By synthesizing current knowledge and prospects, this review aims to enhance understanding and promote advancements in the field, ultimately improving patient outcomes with cardiac arrhythmias.

## Introduction and background

Cardiac arrhythmias are disorders of the heart's electrical system, resulting in abnormal heart rhythms [1]. These abnormalities can manifest as irregular heart rate, rhythm, or conduction pathways. Arrhythmias are broadly classified into two main categories: tachyarrhythmias, characterized by an abnormally fast heart rate (over 100 beats per minute), and bradyarrhythmias, where the heart rate is abnormally slow (under 60 beats per minute) [2]. Subcategories include supraventricular arrhythmias, originating above the ventricles, and ventricular arrhythmias, originating within the ventricles. Common types of arrhythmias include atrial fibrillation (AF), atrial flutter, supraventricular tachycardia (SVT), ventricular tachycardia (VT), and ventricular fibrillation (VF) [2]. Cardiac arrhythmias are prevalent worldwide, affecting millions of people. Atrial fibrillation is the most common type, with an estimated 33 million individuals affected globally. The incidence of arrhythmias increases with age, and they are more common in individuals with underlying cardiovascular diseases such as hypertension, coronary artery disease, and heart failure [3]. The prevalence of arrhythmias is also higher in populations with certain risk factors, including obesity, diabetes, and a sedentary lifestyle. The growing aging population and increasing prevalence of cardiovascular risk factors contribute to the rising incidence of cardiac arrhythmias [3].

Effective management of cardiac arrhythmias is crucial due to their potential to cause significant morbidity and mortality [4]. Untreated arrhythmias can lead to severe complications, such as stroke, heart failure, and sudden cardiac death. For instance, atrial fibrillation is a major risk factor for stroke, increasing the risk by fivefold. Ventricular arrhythmias, particularly ventricular fibrillation, can lead to sudden cardiac arrest, a leading cause of death. Proper management can improve quality of life, reduce the risk of complications, and enhance survival rates [4]. This comprehensive review aims to provide an in-depth examination of current practices and emerging therapies in the management of cardiac arrhythmias. This review seeks to inform healthcare professionals about the most effective approaches to diagnosing and managing these complex conditions by exploring the latest advancements and treatment strategies. The review will cover the pathophysiology, clinical presentation, diagnostic techniques, and current and emerging treatment options, highlighting the evolving landscape of cardiac arrhythmia management. Through this review, we aim to underscore the importance of continued research and innovation in improving patient outcomes and addressing the challenges associated with cardiac arrhythmias.

## Review

Pathophysiology of cardiac arrhythmias

The heart's electrical conduction system is crucial for maintaining a coordinated heartbeat. It comprises several key components: the sinoatrial (SA) node, atrioventricular (AV) node, and bundle of His and Purkinje fibers. The SA node in the right atrium acts as the heart's natural pacemaker, generating electrical impulses that initiate each heartbeat. These impulses spread through the atrial myocardium, causing the atria to contract and push blood into the ventricles [5]. The electrical signal then reaches the AV node, which is briefly delayed to allow for complete ventricular filling. Following this delay, the impulse travels down the bundle of His into the Purkinje fibers, which distribute the electrical signal throughout the ventricles, leading to their contraction. This highly organized conduction system ensures that the heart beats synchronously, allowing efficient blood circulation throughout the body [6]. Arrhythmias can arise from various mechanisms, primarily categorized into abnormal impulse initiation and impulse conduction. Abnormal automaticity occurs when pacemaker cells outside the SA node spontaneously generate electrical impulses, often due to changes in membrane potential. Triggered activity is another mechanism where early or delayed afterdepolarizations lead to additional impulses that disrupt the normal rhythm [7]. Re-entry is a third mechanism characterized by a unidirectional conduction block combined with slowed conduction, allowing an electrical impulse to re-enter and repeatedly excite the same myocardial tissue. These mechanisms can lead to various arrhythmias with clinical implications [7]. Multiple risk factors and underlying conditions can predispose individuals to cardiac arrhythmias. Structural heart diseases, such as coronary artery disease, cardiomyopathy, and valvular heart disease, can alter the heart's anatomy and electrical properties, increasing the likelihood of arrhythmias. Electrolyte imbalances, particularly hypokalemia and hypomagnesemia, significantly affect cardiac excitability and conduction. Certain medications, including antiarrhythmics and stimulants, may also provoke arrhythmias [8]. Lifestyle factors, such as excessive alcohol consumption and recreational drug use, can contribute to arrhythmogenesis. Additionally, congenital heart defects, autonomic nervous system imbalances, and viral infections, such as those seen in coronavirus disease 2019 (COVID-19), have been identified as potential triggers. Understanding these risk factors is crucial for clinicians in identifying at-risk patients and implementing preventive strategies to manage and mitigate the impact of cardiac arrhythmias [8].

Clinical presentation and diagnosis

**Figure 1:**
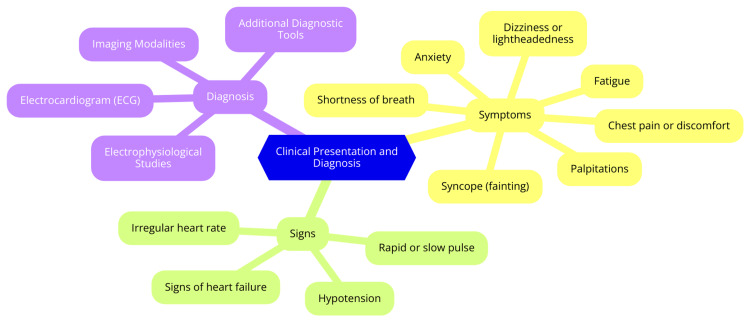
Clinical presentation and diagnosis Image Credit: Dr Anmol Nagpal

Cardiac arrhythmias can present with a wide range of symptoms, including palpitations, dizziness, fainting, chest discomfort, shortness of breath, fatigue, and anxiety. However, it is important to note that some arrhythmias can be asymptomatic and go unnoticed, with the irregular heartbeat detected during a routine physical examination or incidentally during diagnostic testing for other conditions [9]. Healthcare providers utilize various diagnostic tools and techniques to identify the type and cause of cardiac arrhythmia. The electrocardiogram (ECG) is often the first diagnostic test performed when an arrhythmia is suspected, as it records the heart's electrical activity and can help detect abnormal heart rhythms [10]. Holter monitoring, involving a portable ECG device that continuously records the heart's electrical activity for 24 to 48 hours or longer, can help identify intermittent or transient arrhythmias that may not be present during a standard ECG [10]. Event recorders are similar to Holter monitors but are designed to record the heart's activity only when the patient experiences symptoms. The patient activates the device when they feel palpitations, dizziness, or other symptoms, allowing for targeted recording of the arrhythmia [11]. Electrophysiological studies involve inserting catheters into the heart to map the electrical activity and identify the precise location of the arrhythmia, which is crucial for guiding treatment, particularly for catheter ablation procedures [11]. Various imaging techniques, such as echocardiography and cardiac MRI, can provide valuable information about the structure and function of the heart. These tests can help identify underlying heart conditions that may contribute to the development of arrhythmias [12]. Diagnosing a cardiac arrhythmia typically involves a combination of the patient's medical history, physical examination, and the results of diagnostic tests. Healthcare providers will consider the specific symptoms, the duration and frequency of the irregular heartbeats, and any underlying medical conditions to determine the most appropriate treatment plan [12]. Clinical presentation and diagnosis are shown in Figure 1.

Types of cardiac arrhythmias

Cardiac arrhythmias can be categorized into three main groups: supraventricular, ventricular, and bradyarrhythmias. Each group has specific conditions that differ in origin, symptoms, and management strategies [12]. Supraventricular arrhythmias arise from the atria or the AV node and are characterized by abnormal electrical activity affecting the heart's upper chambers. The most common type is AF, marked by rapid and irregular electrical signals in the atria, leading to ineffective contractions. Patients with AF may experience palpitations, fatigue, shortness of breath, and an increased risk of stroke [13]. Management typically involves rate control using medications like beta-blockers, rhythm control with antiarrhythmic drugs, and anticoagulation therapy to prevent thromboembolic events. Another supraventricular arrhythmia is atrial flutter, characterized by rapid but organized electrical activity in the atria, often presenting as a "sawtooth" pattern on an ECG. Symptoms are similar to those of AF, with treatment options including electrical cardioversion, catheter ablation, and rate control medications [14]. SVT encompasses arrhythmias originating above the ventricles, leading to a rapid heart rate. Common types include AV nodal reentrant tachycardia (AVRT) and atrial tachycardia. Patients may experience sudden onset of rapid heart rate, palpitations, dizziness, and sometimes syncope. Management options include vagal maneuvers, medications like adenosine, and catheter ablation for recurrent cases [15].

**Figure 2:**
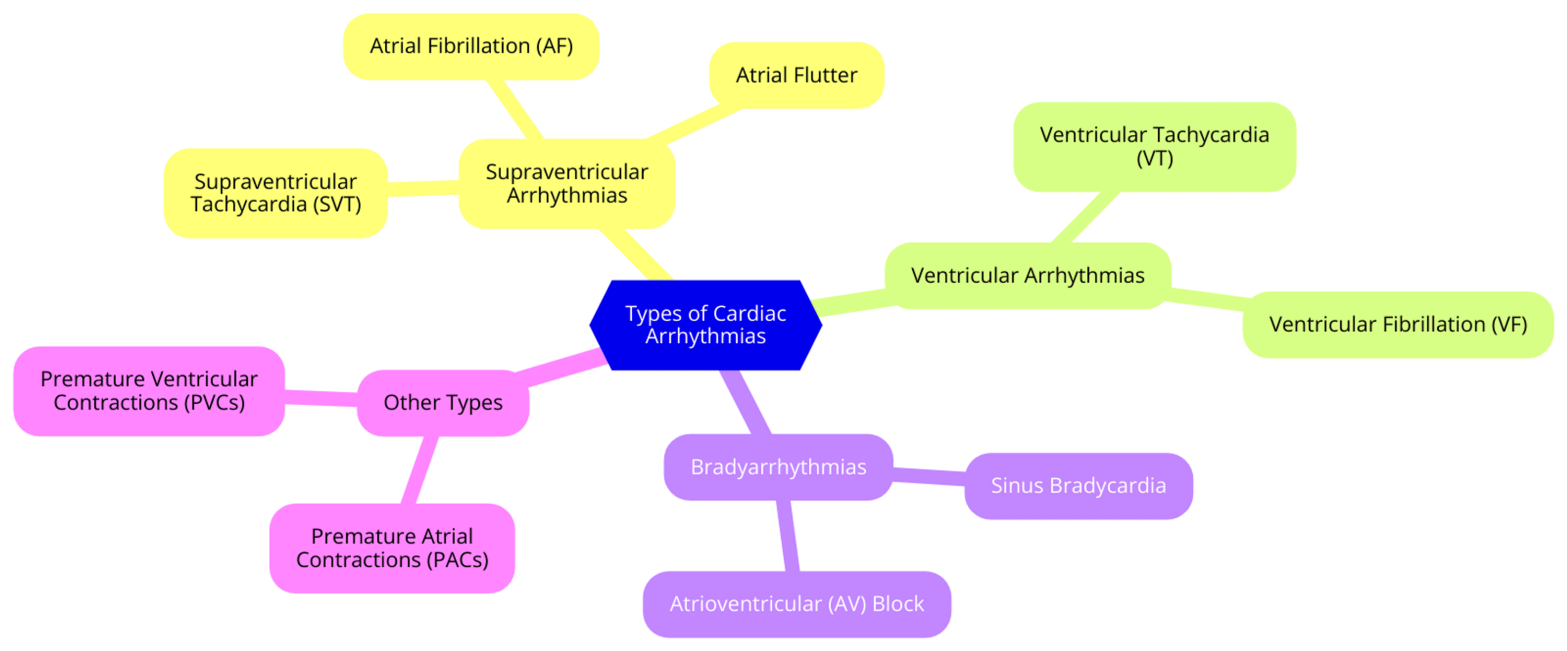
Types of cardiac arrhythmias Image Credit: Dr Anmol Nagpal

Ventricular arrhythmias originate in the ventricles and can be life-threatening. VT is three or more consecutive ventricular beats at a rate exceeding 100 beats per minute. VT can be stable, with the patient remaining conscious, or unstable, leading to loss of consciousness. Symptoms may include palpitations, dizziness, chest pain, or syncope. Management typically involves antiarrhythmic medications, electrical cardioversion, and, in some cases, catheter ablation [16]. VF is a more critical condition characterized by chaotic, disorganized electrical activity in the ventricles, resulting in ineffective contraction and loss of cardiac output. It leads to sudden cardiac arrest, with patients experiencing loss of consciousness and absence of pulse. Immediate cardiopulmonary resuscitation (CPR) and defibrillation are essential for survival in such cases [16]. Bradyarrhythmias are characterized by a heart rate slower than normal, typically defined as less than 60 beats per minute. Sinus bradycardia is a common heart rate that is slower due to decreased sinus node automaticity. Many patients with sinus bradycardia are asymptomatic, but some may experience fatigue, dizziness, or syncope [17]. Treatment may not be necessary unless symptoms are present; if symptomatic, interventions such as atropine or pacing may be required. Another important category within bradyarrhythmias is AV block, occurring when conduction through the AV node is impaired. AV block can be classified into three degrees: first-degree AV block, characterized by a prolonged PR interval with all impulses conducted; second-degree AV block, where some impulses are blocked (Mobitz type I or II); and third-degree AV block, a complete block where no impulses are conducted to the ventricles. Symptoms can range from asymptomatic to syncope, depending on the degree of block, and management may involve monitoring, with more severe cases often requiring pacemaker implantation [17]. Types of cardiac arrhythmias are shown in Figure 2.

Current management strategies

The current management strategies for cardiac arrhythmias include both pharmacological and non-pharmacological treatments. Antiarrhythmic drugs are classified into four main classes (I-IV) based on their mechanism of action. Class I drugs are sodium channel blockers (e.g., lidocaine, flecainide, propafenone), Class II are beta-blockers (e.g., atenolol, metoprolol), Class III are potassium channel blockers (e.g., amiodarone, sotalol, ibutilide), and Class IV are calcium channel blockers (e.g., diltiazem, verapamil). These drugs work by altering the electrical properties of the heart tissue to prevent or terminate arrhythmias [18]. In patients with AF, the primary pharmacological options are rate and rhythm control. Rate control involves using medications like beta-blockers or calcium channel blockers to slow the heart rate. In contrast, rhythm control uses antiarrhythmic drugs (e.g., amiodarone, flecainide) to restore and maintain a normal heart rhythm. The choice between rate and rhythm control depends on factors such as symptom severity, duration of AF, and patient preferences [18]. Non-pharmacological treatments for cardiac arrhythmias include electrical cardioversion, catheter ablation, device therapy, and surgery. Electrical cardioversion uses controlled electric shocks to restore normal heart rhythm in patients with AF or atrial flutter. Catheter ablation involves destroying abnormal heart tissue and causing arrhythmia using heat or cold energy delivered through a catheter [19]. Device therapy includes pacemakers to treat bradycardia and maintain an adequate heart rate and implantable cardioverter-defibrillators (ICDs) for patients at risk of life-threatening ventricular arrhythmias to deliver shocks when needed. Surgical treatments include the Maze procedure, which creates a maze-like pattern of scar tissue to block abnormal electrical signals in AF, and surgical ablation performed during open-heart surgery to treat arrhythmias [19].

Emerging therapies

Managing cardiac arrhythmias is evolving rapidly, with several emerging therapies showing promise for improved outcomes. Among these, advances in catheter ablation techniques stand out as particularly significant. Catheter ablation has become a cornerstone treatment for various arrhythmias, particularly atrial fibrillation. Cryoablation, which uses extreme cold to create lesions in cardiac tissue, is gaining traction as a less invasive alternative to traditional radiofrequency ablation [20]. The cryoballoon technique, in particular, has demonstrated excellent efficacy in isolating pulmonary veins. Laser ablation technology has evolved, with second-generation laser balloons offering enhanced tissue contact and visibility, thereby reducing procedural times and improving outcomes. Robotic-assisted ablation systems enhance precision, allowing for more accurate catheter manipulation during complex procedures, which may lead to better safety and efficacy [21]. In parallel with advancements in catheter ablation, developing novel antiarrhythmic drugs is a key area of research. While traditional antiarrhythmics remain important, new agents are being investigated for their potential to improve safety and efficacy. One such agent, vernakalant, is an atrial-selective antiarrhythmic that blocks multiple ion channels and has shown promise in converting recent-onset atrial fibrillation to sinus rhythm with a favorable side effect profile [22]. Additionally, researchers are exploring antiarrhythmic peptides-short sequences of amino acids that can modulate gap junction and ion channel functions in new therapeutic avenues without the proarrhythmic effects seen in some conventional medications [22].

**Figure 3:**
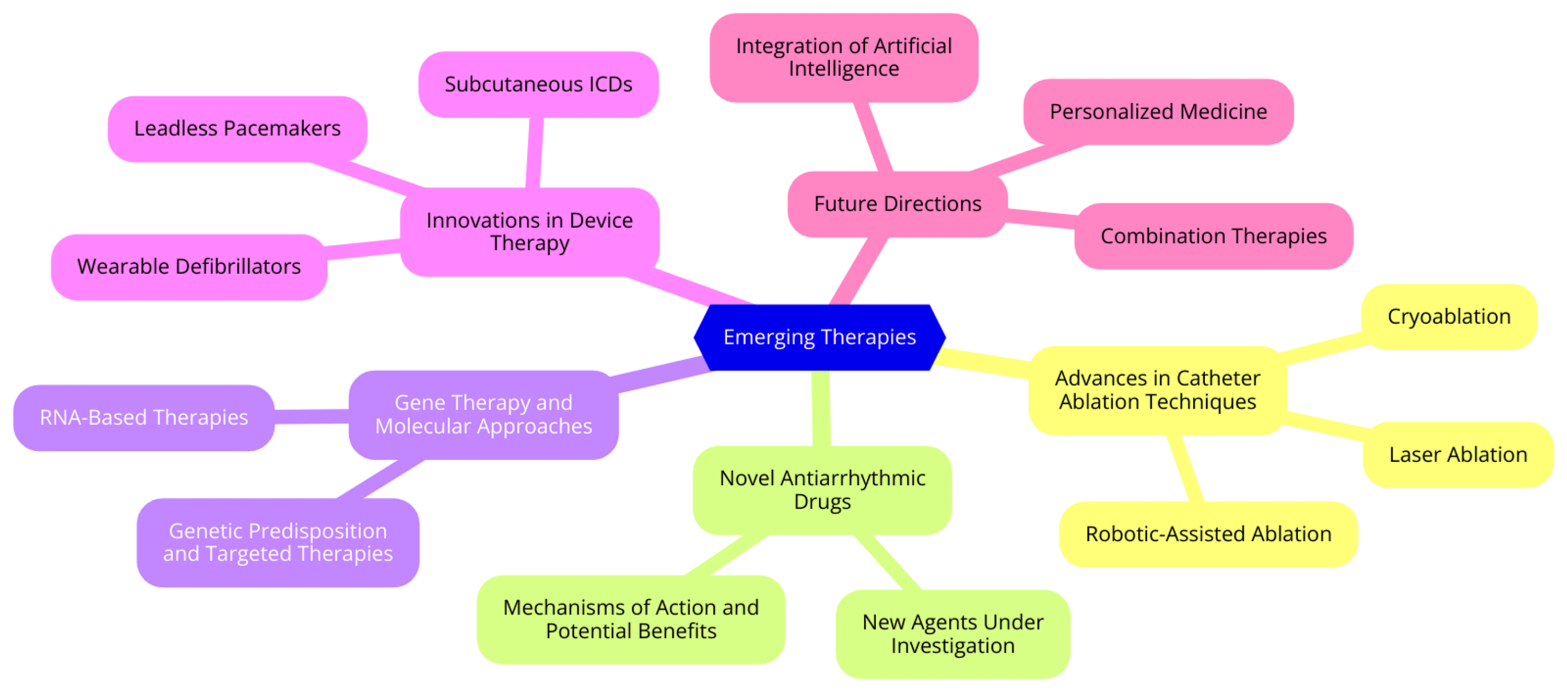
Emerging therapies Image Credit: Dr Anmol Nagpal

Gene therapy and molecular approaches are also emerging as innovative strategies in treating arrhythmias. Advances in genetic testing allow for the identification of genetic predispositions to certain arrhythmias, enabling targeted screening and preventive strategies [23]. Furthermore, gene editing technologies, such as CRISPR/Cas9, can potentially correct genetic defects associated with inherited arrhythmia syndromes. RNA-based therapies, including small interfering RNAs (siRNAs) and antisense oligonucleotides, offer a novel approach to modulating gene expression and ion channel function, which could significantly impact the management of arrhythmias [23]. Lastly, innovations in device therapy are transforming the landscape of arrhythmia management. Leadless pacemakers represent a significant advancement, as these self-contained devices are implanted directly into the right ventricle, eliminating the need for leads and reducing complications associated with transvenous systems [24]. Subcutaneous ICDs are another innovative option, providing an alternative for patients who cannot tolerate traditional transvenous leads. Additionally, wearable defibrillators, designed as vest-like devices, can detect and treat life-threatening arrhythmias in high-risk patients, offering temporary protection while awaiting more definitive treatment [24]. Emerging therapies are shown in Figure 3.

Special populations and considerations

Pediatric arrhythmias present unique challenges and considerations, as they can manifest differently than in adults. Common types include long QT syndrome, which can lead to fast, erratic heartbeats and potentially serious complications if left untreated. Management of this condition often involves medications, pacemakers, or ICDs [25]. Another notable condition is Wolff-Parkinson-White syndrome (WPW), characterized by an extra electrical pathway in the heart that results in episodes of rapid heart rates. Treatment for WPW may include medications or catheter ablation to eliminate the abnormal pathway. Additionally, bradycardia, or a slower-than-normal heart rate, may occur in children and require monitoring or the implantation of a pacemaker if the child exhibits symptoms [26]. Atrial fibrillation and flutter, while rare in the pediatric population, can occur in children with congenital heart defects or those who have undergone heart surgery, necessitating treatment that typically involves medications or cardioversion. While most pediatric arrhythmias are benign, some can be life-threatening, highlighting the importance of careful monitoring and intervention by pediatric cardiologists [27]. Elderly patients are particularly susceptible to arrhythmias due to age-related changes in the heart's electrical system and the presence of comorbidities such as hypertension and heart failure. Atrial fibrillation is the most prevalent arrhythmia in older adults, significantly increasing the risk of stroke and heart failure [28]. Management of AF in this population often involves anticoagulation therapy, along with strategies for rate or rhythm control. Bradyarrhythmias are also common in the elderly, arising from degenerative changes in the heart's conduction system. In cases of symptomatic bradycardia, pacemaker implantation may be necessary to restore a normal heart rate. Additionally, elderly patients may experience exercise-induced arrhythmias, which complicate their management due to the potential for underlying heart disease. Thus, a comprehensive approach is essential for effectively managing arrhythmias in older adults [29].

Pregnancy introduces physiological changes that can significantly affect heart rhythm, leading to arrhythmias. Increased blood volume and cardiac output during pregnancy can exacerbate pre-existing arrhythmias or lead to new-onset conditions, such as atrial fibrillation, particularly in women with structural heart issues [30]. Hormonal fluctuations throughout pregnancy can also influence heart rhythm. The management of arrhythmias during pregnancy requires a careful balance of maternal and fetal health, often involving a multidisciplinary team to tailor treatment strategies. It is crucial to consider both the safety of the mother and the developing fetus when determining the most appropriate course of action [31]. Athletes may experience exercise-induced arrhythmias due to the heightened demands placed on the heart during physical activity. Endurance athletes, in particular, often develop a lower resting heart rate and may exhibit benign conditions such as bradycardia or sinus arrhythmia. While most exercise-related arrhythmias are harmless, there is an inherent risk of serious conditions, such as ventricular tachycardia or fibrillation, especially in athletes with underlying heart conditions [32]. Therefore, monitoring and assessment are critical. Athletes should undergo thorough cardiovascular evaluations, including ECGs and possibly echocardiograms, to identify potential risks before engaging in high-intensity training. This proactive approach helps ensure the safety and well-being of athletes as they pursue their fitness goals [33].

Future directions and research

The future of cardiac arrhythmia management is poised for significant advancements, particularly through personalized medicine. This approach emphasizes tailoring treatment strategies to individual patients based on their unique genetic, environmental, and lifestyle factors. As genetic testing becomes more accessible and affordable, it will enable healthcare providers to identify specific genetic variants associated with arrhythmias [34]. This knowledge can guide the selection of antiarrhythmic medications and interventions most likely effective for each patient. Moreover, integrating genetic data with electronic health records can facilitate proactive risk stratification, allowing clinicians to identify patients at higher risk for arrhythmias and implement preventive measures early in life [35]. Integrating artificial intelligence (AI) and machine learning (ML) into arrhythmia management represents another promising frontier. AI and ML algorithms can analyze vast amounts of data from ECGs, Holter monitors, and implantable devices to improve diagnostic accuracy and speed [36]. These technologies can also develop predictive models that identify high-risk patients for arrhythmias, enabling timely interventions. Additionally, AI can optimize catheter ablation strategies by analyzing patient-specific data to enhance procedural outcomes. As these technologies evolve, they will provide a more nuanced understanding of arrhythmias, leading to more precise and personalized treatment plans [37].

Long-term outcomes and quality of life following arrhythmia interventions are critical for future research. Evaluating the impact of various treatment modalities, such as medications, catheter ablation, and implantable devices, on patients’ symptoms, functional status, and overall well-being is essential [38]. By identifying predictors of favorable outcomes, clinicians can better tailor therapies to individual needs. Furthermore, incorporating patient-reported outcome measures will ensure that the patient's voice is central to evaluating treatment effectiveness, ultimately leading to improved quality of life for those living with arrhythmias [39]. Addressing disparities in access to arrhythmia care is a pressing challenge that must be tackled to ensure equitable healthcare for all patients. Expanding telehealth services and remote monitoring can help reach underserved populations, providing them access to necessary care and resources. Developing culturally tailored patient education materials and shared decision-making tools can also empower patients from diverse backgrounds to engage actively in their care [40]. Promoting diversity in clinical trials is also vital to ensure precision medicine approaches apply to a broad range of populations. By advocating for policies that improve insurance coverage and affordability of advanced diagnostics and therapies, we can work towards eliminating barriers to care and enhancing outcomes for all individuals affected by cardiac arrhythmias [41].

## Conclusions

In conclusion, managing cardiac arrhythmias is a dynamic and rapidly evolving field, encompassing various diagnostic and therapeutic approaches. From traditional pharmacological treatments and electrical interventions to cutting-edge techniques like catheter ablation and novel device therapies, the landscape of arrhythmia management continues to advance, driven by ongoing research and technological innovations. Emerging therapies, including gene therapy and AI-driven diagnostics, hold promise for more personalized and effective treatment strategies. The burden of arrhythmias on global health necessitates a concerted effort to optimize existing treatments and develop new interventions to reduce morbidity and mortality associated with these conditions. Continued research, innovation, and clinical trials are essential to address the challenges in arrhythmia management, improve patient outcomes, and enhance the quality of life for those affected by these potentially life-threatening disorders.
